# Factors contributing to mitogenome size variation and a recurrent intracellular DNA transfer in *Melastoma*

**DOI:** 10.1186/s12864-023-09488-x

**Published:** 2023-07-01

**Authors:** Shuaixi Zhou, Xueke Zhi, Runxian Yu, Ying Liu, Renchao Zhou

**Affiliations:** State Key Laboratory of Biocontrol and Guangdong Provincial Key Laboratory of Plant Resources, School of Life Sciences, Sun Yat-Sen University, Guangzhou, 510275 China

**Keywords:** Mitogenome, Size variation, Intracellular DNA transfer, Horizontal DNA transfer, Melastoma

## Abstract

**Background:**

Mitogenome sizes of seed plants vary substantially even among closely related species, which are often related to horizontal or intracellular DNA transfer (HDT or IDT) events. However, the mechanisms of this size variation have not been well characterized.

**Results:**

Here we assembled and characterized the mitogenomes of three species of *Melastoma*, a tropical shrub genus experiencing rapid speciation. The mitogenomes of *M. candidum* (Mc), *M. sanguineum* (Ms) and *M. dodecandrum* (Md) were assembled to a circular mapping chromosome of 391,595 bp, 395,542 bp and 412,026 bp, respectively. While the mitogenomes of Mc and Ms showed good collinearity except for a large inversion of ~ 150 kb, there were many rearrangements in the mitogenomes between Md and either Mc or Ms. Most non-alignable sequences (> 80%) between Mc and Ms are from gain or loss of mitochondrial sequences. Whereas, between Md and either Mc or Ms, non-alignable sequences in Md are mainly chloroplast derived sequences (> 30%) and from putative horizontal DNA transfers (> 30%), and those in both Mc and Ms are from gain or loss of mitochondrial sequences (> 80%). We also identified a recurrent IDT event in another congeneric species, *M. penicillatum*, which has not been fixed as it is only found in one of the three examined populations.

**Conclusions:**

By characterizing mitochondrial genome sequences of *Melastoma*, our study not only helps understand mitogenome size evolution in closely related species, but also cautions different evolutionary histories of mitochondrial regions due to potential recurrent IDT events in some populations or species.

**Supplementary Information:**

The online version contains supplementary material available at 10.1186/s12864-023-09488-x.

## Introduction

The mitogenomes of seed plants vary considerably in size [1–5], ranging from 65.9 kb in a hemiparasitic plant *Viscum scurruloideum* [6] to 11.3 Mb in *Silene conica* [5] and 11.7 Mb in *Larix sibirica* [7]. Dramatic variations in mitogenome size have also been observed between closely related species within a family [8, 9], and even between species within a single genus [5, 6, 10]. For example, species of *Silene* have more than 40-fold difference in mitogenome size, with normal sizes of 0.43 Mb and 0.25 Mb for *S. vulgaris* and *S. latifolia*, while huge sizes of 6.7 Mb and 11.3 Mb for *S. noctiflora* and *S. conica* [5]. Another example is from *Viscum*, in which *V. album* has a mitogenome size of 565.5 kb [10], while *V. scurruloideum* possesses the smallest mitogenome size of 65.9 kb among sequenced seed plants [6].

Although the content of mitochondrial protein-coding genes and tRNAs vary markedly among seed plants [2, 11, 12], variable intergenic regions are largely responsible for much of the mitogenome size variation. The intergenic regions usually contain considerable amounts of integrated foreign DNA originating from the plastid and nuclear genomes via intracellular DNA transfer (IDT) or from other species (mainly plants) via horizontal DNA transfer (HDT) [2, 13–17]. In addition, some plants can integrate DNA originating from the plasmids [18, 19], bacteria and viruses [13, 20]. The other dominant contributor to variable intergenic regions is repetitive sequences. Repetitive sequences constitute a variable and often large component of mitochondrial genomes of seed plants [13, 21–23], although no correlation was found between repetitive DNA and genome size [2, 21, 24]. The proliferation of repetitive DNA may have contributed to expansions in intergenic regions, like numerous copies of repetitive Bpu sequences in the mitogenome of *Cycas taitungensis* [25].

Intergenic regions in the mitogenomes of seed plants always exhibit rapid sequence turnover [5, 26–29], making some and even a vast majority of them non-alignable between distantly and even closely related plants. Comparative analysis among closely related species within a genus or different lines within a species may be advantageous for comparing sequences of their intergenic regions and provide insights into mitogenome size evolution. For example, roughly one quarter of variation in genome size among five lines of maize owes almost exclusively to differences in repeat content [26] and about 18% of the unique regions distinguishing the mitogenomes of fertile and cytoplasmic male sterility (CMS) cytotypes of *Beta vulgaris* results from nuclear insertions [30]. Mitogenome sizes of two individuals of *Silene noctiflora* differ by 410 kb, resulting from the presence or absence of 19 entire chromosomes that lack any identifiable genes or contain only duplicate gene copies [31]. In four species of *Silene*, 6.7–40.8% of their mitogenomes are made up of dispersed repeats, and less than 1% of the intergenic regions contain sequences of nuclear or plastid origin [5].

A major challenge in plant mitogenome evolution is to precisely identify the intrinsic and extrinsic sources of the vast amounts of intergenic sequences [2]. For instance, although some of line-specific sequences in *Beta vulgaris* could be traced to mitochondrial plasmids and recent chloroplast- or nucleus-derived insertions, 70% of the unique sequence was unidentifiable [32]. In two species of *Silene* with huge mitogenomes, 85% of the intergenic sequences lacks detectable homology with any known DNA sequence [5]. These unidentifiable sequences might have originated from their own nuclear genomes, however, nuclear genome sequences from multiple species of a single genus or multiple individuals of a single species, are usually unavailable to test this hypothesis.

*Melastoma,* a shrub genus of about 100 species distributed in tropical Asia and Oceania [33, 34], may be a good system to study mitogenome size evolution, because species of this genus diversified very rapidly and have been formed in the past 1 ~ 2 million years [35]. Moreover, three species of this genus, *M. candidum*, *M. sanguineum* and *M. dodecandrum*, have available nuclear genome sequences [36–38], making it feasible to investigate the contribution of nuclear genome to mitogenome size change among them. The three well-separated species exhibit different morphological traits: both *M. candidum* and *M. sanguineum* are erect shrubs, but the former has long, soft hairs on leaf, and appressed scales on twig and hypanthium (Fig. S1A), while the latter possess no hairs on leaf, and spreading bristles on twig and hypanthium (Fig. S1B); while *M. dodecandrum* is the only creeping species in this genus, with very short, sparse hairs on leaf, twig and hypanthium (Fig. S1C). In habitat, *M. candidum* is a light demanding opportunist, often occurring in open fields, grasslands and roadsides. In contrast, *M. sanguineum* prefers shady environments, and is usually found in the edge of forest understory. *M. dodecandrum* is usually found in grasslands and roadsides, but shows some extent of shade tolerance. Here we assembled and characterized the complete mitogenomes of the three *Melastoma* species (one for each), to investigate the factors that contribute to their mitogenome size variation. We found intracellular DNA transfer, originating from chloroplast genomes, and small-scale gain and loss of mitochondrial sequences can largely account for mitogenome size variation among them. By comparing one IDT region in multiple species of *Melastoma*, we also identified a recurrent IDT event, which occurred, but has not been fixed in another species of this genus, suggesting the process of IDT is still ongoing.

## Materials and methods

### Plant sampling, DNA isolation and Illumina sequencing

For mitogenome assembly, one individual each of the three species of *Melastoma* (*M. candidum, M. sanguineum,* and *M. dodecandrum*) was sampled from different locations in China. To test if recurrent IDT events occurred, one individual each of five other species (*M. imbricatum, M. dendrisetosum, M. penicillatum, M. normale* and *M. malabathricum*) was sampled. To test if the identified recurrent IDT event exist in multiple populations of *M. penicillatum*, five individuals each from three populations, namely, Diaoluoshan, Wuzhishan and Jianfengling, all from central Hainan, China, were sampled. Our collection work complies with the laws of the People's Republic of China and has been permitted by the local departments of forestry. Dr. Renchao Zhou had formally authenticated the collected plant materials. Details of sampling information were shown in Table S1. Voucher specimens have been deposited in the Herbarium of Sun Yat-sen University (SYS). Fresh leaves were collected and then used for DNA extraction with a HiPure Plant DNA Mini Kit (Magen, Guangzhou, China). For each of the three species (*M. candidum, M. sanguineum,* and *M. dodecandrum*), a shotgun DNA library with an insert size of 350 bp was constructed and then sequenced on an Illumina Hiseq X Ten platform with paired-end reads of 150 bp (NCBI accession numbers: SRR22574046, SRR23109727 and SRR19183021)*.* About 8 Gb Illumina sequences were obtained for each species. PacBio reads of the three species were obtained in previous genome sequencing projects [36–38] (NCBI accession numbers: SRR22574047, SRR23109726 and SRR23112531). See Availability of Data and Materials for detailed information.

### Mitochondrial genome assembly and annotation

Novoplasty v2.7.2 [39] was first used to assemble the mitogenome of *M. candidum* with Illumina reads. The mitochondrial *cox1* gene of *Eucalyptus grandis* (GenBank accession number NC_040010.1) was selected as the seed and the parameter k-mer was set to 37. Ten contigs with a total length of ~ 600 kb, were obtained. PacBio long reads of *M. candidum* were corrected in CANU v1.8 [40] with the following parameters: genomeSize = 300 m and corOutCoverage = 50. The corrected PacBio long reads were mapped to these 10 contigs using Blasr v5.3.3 [41] to choose mitogenome-derived reads with the parameters minAlnLength = 2000 and minPctSimilarity = 80. The extracted PacBio reads were supplied to CANU v1.8 for further assembly. Contigs yielded by CANU were regarded as the reference, and the assembly was carried out several times until the longest contig was stably 387,487 bp. To circularize the contig, we used the sequences of *rpl2* on one end and *nad5* on the other end of the contig (annotated by GeSeq, [42]) as seeds to execute contig extension in Novoplasty with Illumina reads. This yields a 4,108 bp contig that could connect two ends, and thus circularizes the draft mitogenome of *M. candidum*. Due to the relatively high sequencing error rate of PacBio reads, Pilon v1.3 [43] was used to polish the draft mitogenome with Illumina reads. For *M. sanguineum* and *M. dodecandrum*, we used the mitogenome sequence of *M. candidum* as the initial reference to choose mitogenome-derived PacBio reads, and assembled their mitogenomes using the same method as that used for *M. candidum*. The mitogenome sequences of the three species were deposited in GenBank under accession numbers MZ490595, MZ490596 and MZ490597.

To identify intracellular DNA transfer (IDT) from chloroplast genome to mitogenome, we searched the mitochondrial genome sequence of each species against its chloroplast genome sequence using Blastn with default parameters except -perc_identity set to 85, and hits over 90 bp in length were recorded. The result for each species was shown in a circular diagram using Circos [44].

The protein-coding genes in the three mitogenomes were annotated using GeSeq [42] and further manual adjustment was conducted when necessary. Annotation of rRNA and tRNA gens was carried out using RNammer [45] and tRNAscan-SE [46] with the organelle option, respectively. Dispersed repeats were identified with ROUSfinder.py [47] with default parameters except for the minimal repeat size set to 30. For repeat pairs larger than 100 bp, we followed the method of Dong et al. [21] with a custom script to examine the recombination rates.

The three mitogenomes were compared using MUMmer3.23 [48] to detect nucleotide substitutions and indels between them. Synonymous nucleotide substitution rates for mitochondrial protein genes were calculated by MEGA6, using the Kumar model [49] for each sequence pair. Substitution rates in noncoding regions were calculated by MEGA6, using Kimura’s two-parameter model [50]. To calculate the divergence time between *M. candidum* and *M. dodecandrum*, we downloaded mitochondrial protein-coding sequences of *Eucalyptus grandis* (GenBank accession number NC_040010.1), a related species from the same order Myrtales, to calculate the synonymous nucleotide substitution rate between *Melastoma* and *Eucalyptus.*

### Comparison of non-alignable sequences in the mitogenomes of the three species

Pairwise synteny analysis for the three mitogenomes was performed with Mauve v20150226 [51], to identify non-alignable regions. We inferred the origins of the non-alignable regions using a procedure shown in Fig. S2. Specifically, the non-alignable regions were first searched against the chloroplast genome sequence using Blastn with default parameters except -perc_identity set to 85, to identify IDT from chloroplast genome and hits were recorded. These non-alignable regions were then blast searched against each of the three mitochondrial genomes with the same parameter to determine whether they belong to one of the three potential scenarios or not: 1) when a region has more copies in one species than the other species of the species pair, then it is an intragenomic duplication; 2) when a region is absent in the other species of the species pair, but present in another species, it suggests gain or loss of this region in different species of *Melastoma*; and 3) when a region is not found in two other species, it was further searched against the nr database in GenBank and also their own nuclear genomes using Blastn with default parameters except -perc_identity set to 85, to infer the possible origins of these sequences. The nuclear genomes of the three species were available in the website http://evolution.sysu.edu.cn/Sequences.html. The identified intragenomic duplications were shown in the syntenic diagram using Circos.

### Characterization of a recurrent IDT event in *M. penicillatum*

We identified a recurrent IDT event in the mitogenome of *Melastoma penicillatum*, another species in *Melastoma*, by PCR amplification and sequencing an IDT region in multiple species of *Melastoma* from China. The individual of *M. penicillatum* was sampled from Jianfengling, Hainan, China. We designed mitochondrial-specific primers to amplify the IDT regions in the mitogenomes and also chloroplast-specific primers to amplify the corresponding region in the chloroplast genomes of these species for comparison. These primers were designed using the online primer design tool- Primer3_masker [52]. PCR was conducted using KOD One PCR Master Mix (TOYOBO, Osaka, Japan) with the following PCR protocol: pre-denaturation at 98℃ for 3 min, then 98℃ for 10 s, 49℃for 5 s, 68℃for 25 s for 30 cycles, finally extended at 68℃ for 5 min. PCR products were purified and then sequenced using Sanger chemistry with the PCR amplification primers and some internal primers (Table S2). Phylogenetic analysis was carried out to verify the recurrent IDT event in the mitochondrial genome of *M. penicillatum.* Sequences were aligned using MAFFT [53] and the maximum likelihood tree was built with RAxML [54]. To test whether this recurrent IDT event has been fixed in this species or not, we sequenced this region in five individuals from each of the three populations, Diaoluoshan, Wuzhishan and Jianfengling.

## Results and discussion

### Mitogenome size, structure and content of three species of *Melastoma*

**Fig. 1:**
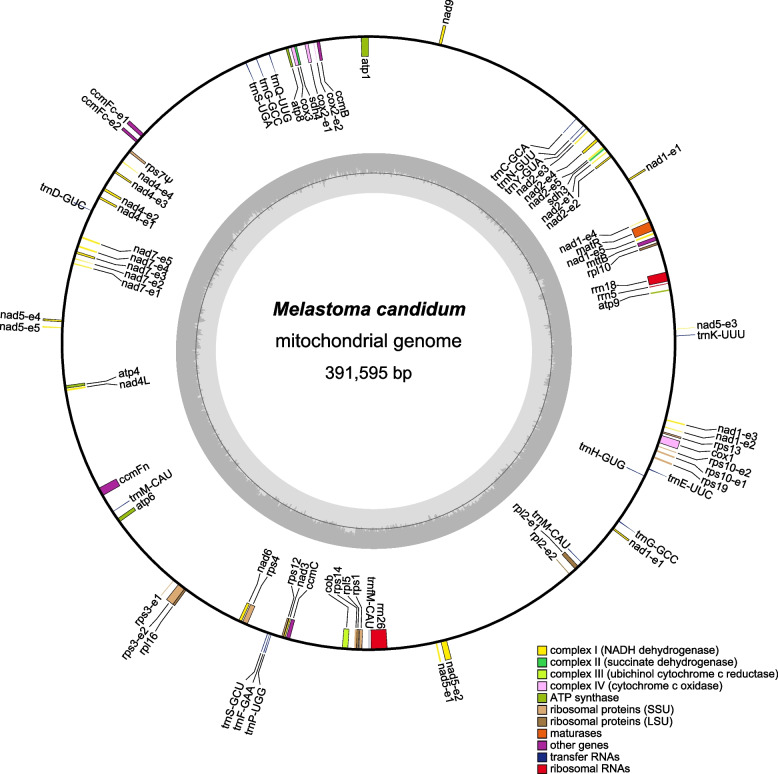
Gene map of the *Melastoma candidum* mitogenome. Chloroplast genome derived genes were not shown in this figure. Pseudogenes are marked with “Ψ”. See Fig. S3 and S4 for gene maps of the *M. sanguineum* and *M. dodecandrum* mitogenomes, respectively

We assembled the mitogenomes of three species of *Melastoma, M. candidum*, *M. sanguineum*, and *M. dodecandrum*, using both Illumina and PacBio reads. All three species have a circular-mapping chromosome with their mitogenome sizes of 391,595 bp, 395,542 bp and 412,026 bp, and overall GC contents of 44.36%, 44.37% and 44.18%, respectively. Both the sizes and GC contents are typical of most angiosperm mitogenomes [2, 21]. The three species have highly similar mitochondrial gene contents, each contain 38 unique protein-coding, 14 tRNA and 3 rRNA genes (Fig. 1). The only difference is one more copy of *atp8* existing in the *M. dodecandrum* mitogenome due to segmental duplication. Of 41 protein-coding genes inferred to have been present in the mitochondrial genome of seed plant common ancestor [2], two genes (*rps2* and *rps11*) have been lost and one gene (*rps7*) has been pseudogenized in all three species. The three mitogenomes each contain 24 group II introns (19 *cis*-spliced and five *trans*-spliced) (Table S3). Moreover, all three species possess 27 protein-coding genes (including truncated and pseudogenized genes), 4 rRNAs and 13 tRNAs resulting from IDT from chloroplast genome, while *M. dodecandrum* has one additional IDT region of ~ 8 kb, which contains 10 other protein-coding genes.

In the mitogenomes of *M. candidum*, *M. sanguineum* and *M. dodecandrum*, 76, 79 and 79 dispersed repeats > 30 bp in length were identified, covering 3.52%, 3.81% and 3.57% of their mitogenomes, respectively (Table 1). The repeat contents in the three *Melastoma* species approach the lower bound of those in angiosperms [2, 21]. Among all repeat pairs larger than 100 bp in the three species, the frequencies of repeat-mediated recombination were zero or very low (Table S4). These low recombination frequencies were comparable with those found in *Nympheae* and *Monsonia* [1, 21], but much lower than those found in *Aeginetia* and *Viscum* [6, 55].

**Table 1:** Summary of the mitogenomes of three *Melastoma* species

Species	*M. candidum*	*M. sanguineum*	*M. dodecandrum*
Genome size (bp)	391,595	395,542	412,026
GC content	44.36%	44.37%	44.18%
Number of mitochondrial protein coding genes (pseudogenes)	38(1)	38(1)	38(1)
Number of chloroplast derived protein coding genes (pseudogenes)	13(14)	12(15)	24(13)
Number of introns	24	24	24
Number of repeat units	76	79	79
Length of the largest repeat (bp)	712	592	878
Repeat content	3.52%	3.81%	3.57%

### Intracellular DNA transfer in the three mitogenomes

**Fig. 2:**
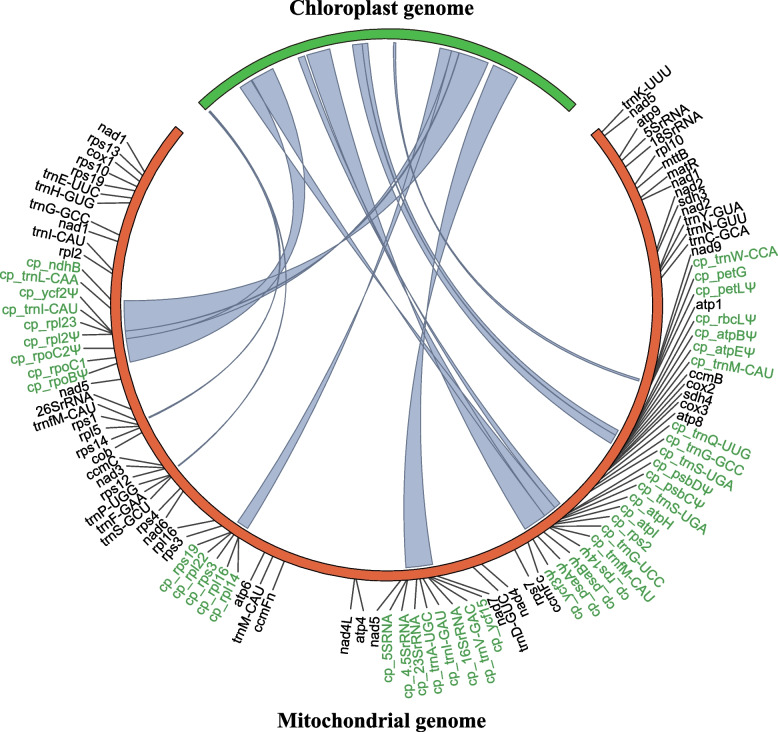
Intracellular transferred DNA from chloroplast genome to the mitochondrial genome of *Melastoma candidum*. Chloroplast genome (one of the IRs excluded here) is colored in green and mitochondrial genome is in orange with annotation information. Chloroplast-derived genes are marked in green and pseudogenes are marked with “Ψ”. Pale blue lines within the circle represent transferred regions from the chloroplast genome. See Fig. S5 and S6 for relevant information in *M. sanguineum* and *M. dodecandrum*, respectively

Synteny analysis between chloroplast and mitochondrial genomes of *M. candidum*, *M. sanguineum* and *M. dodecandrum* revealed the transferred chloroplast DNA via IDT and their distribution in the mitogenomes (Fig. 2). Totally, 53.5 kb in each of *M. candidum* and *M. sanguineum* mitogenomes and 62.5 kb in the *M. dodecandrum* mitogenome were chloroplast derived, and they represent 13.67%, 13.53% and 15.16% of their mitogenomes, respectively. Chloroplast-derived DNA is frequent in angiosperms [2, 56] and its amount varied widely, from 2 ~ 3 kb in *Arabidopsis* and *Vigna* to 113 kb in *Cucurbita* [8, 13, 57]. Thus the proportion of chloroplast-derived DNA in the mitogenomes of *Melastoma* species is moderate. All chloroplast-derived DNA (> 100 bp in length) in the mitogenomes of *M. candidum* and *M. sanguineum* are shared, while *M. dodecandrum* possesses one additional large region (~ 8 kb) and two additional small regions (123 bp and 612 bp) unique to *M. dodecandrum*. This suggests most IDTs should have occurred in the common ancestor of the three species. In line with previous studies [2, 58, 59], most of the protein-coding genes transferred from the chloroplast genome have experienced the process of pseudogenization. Of the 27 chloroplast-derived protein-coding genes, 14 were pseudogenized in both *M. candidum* and *M. sanguineum* due to frameshift indels or truncated coding regions (Fig. 2). The *ndhB* copy has the intact open reading frame in *M. candidum*, but has been pseudogenized in *M. sanguineum* because of substantial truncation. In *M. dodecandrum*, 13 of the 27 chloroplast-derived protein-coding genes shared with two other species are pseudogenes, while all genes but *psbB* in the unique 8 kb region are intact, suggesting that the transfer of the unique region should be relatively young.

### Mitogenome divergence between the three species

**Fig. 3:**
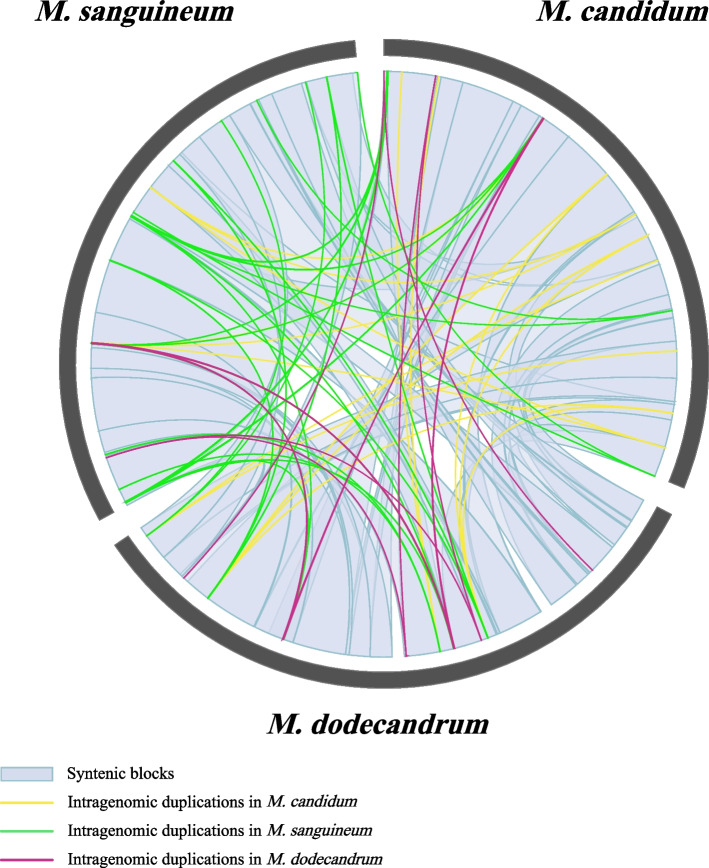
Syntenic blocks and intragenomic duplications among the mitogenomes of three *Melastoma* species. Syntenic blocks are colored with light blue. Intragenomic duplications of short non-alignable regions in the mitogenomes of *M. candidum*, *M. sanguineum* and *M. dodecandrum* are colored with yellow, green and magenta, respectively

**Fig. 4:**
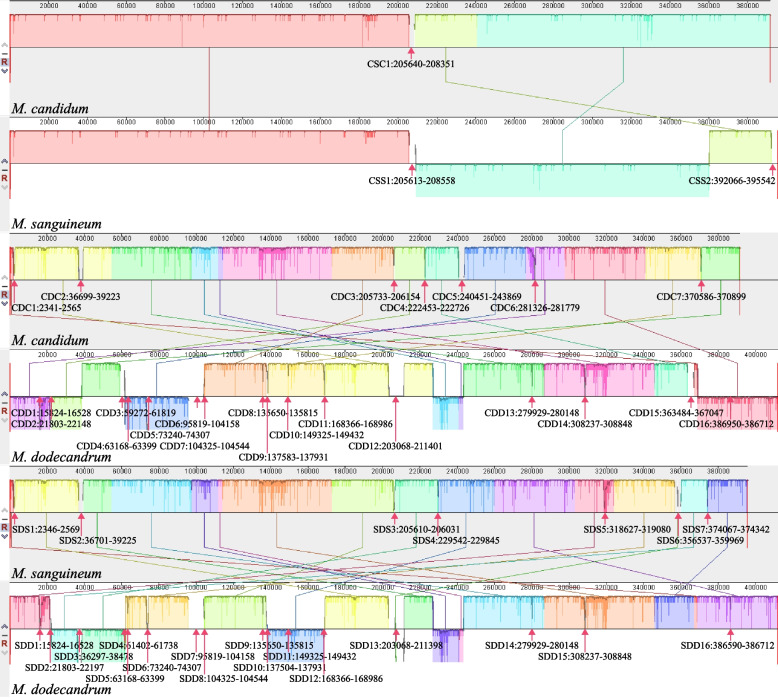
Pairwise MAUVE analysis of the mitogenomes of three *Melastoma* species. Pairwise local syntenic blocks were shown in the same color. The non-alignable mitochondrial regions were coded by the abbreviations of species pair, followed by species name and region number and marked by arrows with their location in the mitogenome. For example, CS and C1 in CSC1 means species pair *M. candidum* (C) and *M. sanguineum* (S), and region 1 in *M. candidum*, respectively

**Fig. 5:**
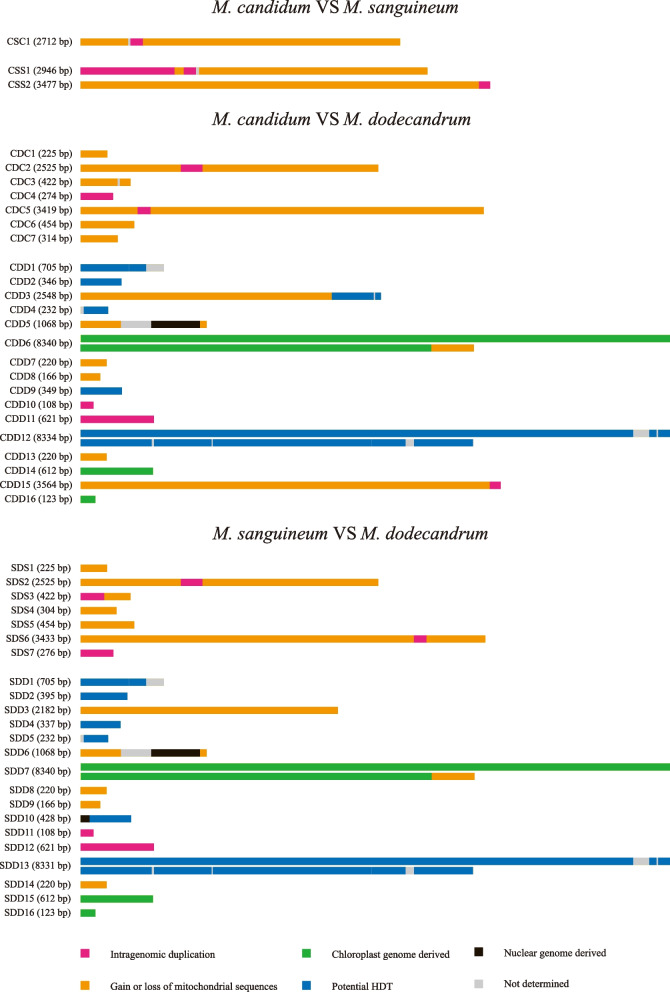
Inferred origins of non-alignable mitochondrial regions identified by pairwise comparison in three *Melastoma* species. The non-alignable mitochondrial regions were coded by the abbreviations of species pair, followed by species name and region number. For example, CS and C1 in CSC1 means species pair *M. candidum* (C) and *M. sanguineum* (S), and region 1 in *M. candidum*, respectively

While the mitogenomes of *M. candidum* and *M. sanguineum* show relatively good collinearity except for a large inversion (see below), there are numerous rearrangements in the mitogenomes between *M. dodecandrum* and either *M. candidum* or *M. sanguineum* (Figs. 3 and 4). Non-alignable sequences in the three species pairs include intragenomic duplications, IDT from the chloroplast/nuclear genomes, gain or loss of mitochondrial regions and potential horizontal DNA transfer (Table 2; Fig. 5).

**Table 2:** Summary of non-alignable regions and their inferred origins in the mitogenomes of three *Melastoma* species

Species pair^a^	Non-alignable regions							
	**Number**	**Inferred origins**^**b**^						
		**CP**	**ID**	**GL**	**PH**	**NU**	**ND**	**Total**
***MC* VS *MS***	1 in MC	0	108 bp (3.98%)	2587 bp (95.39%)	0	0	17 bp (0.63%)	2712 bp (100%)
	2 in MS	0	995 bp (15.49%)	5404 bp (84.14%)	0	0	24 bp (0.37%)	6423 bp (100%)
***MC* VS *MD***	7 in MC	0	569 bp (7.45%)	7047 bp (92.32%)	0	0	17 bp (0.22%)	7633 bp (100%)
	16 in MD	8713 bp (31.62%)	821 bp (2.98%)	6968 bp (25.39%)	9937 bp (36.06%)	415 bp (1.51%)	702 bp (2.55%)	27,556 bp (100%)
***MS* VS *MD***	7 in MS	0	769 bp (10.07%)	6869 bp (89.93%)	0	0	0	7638 bp (100%)
	16 in MD	8713 bp (36.17%)	729 bp (3.03%)	3546 bp (14.72%)	9923 bp (41.19%)	490 bp (2.03%)	687 bp (2.85%)	24,088 bp (100%)

^a^*MC M. candidum*, *MS M. sanguineum*, *MD M. dodecandrum*

^b^*CP* Chloroplast genome derived, *ID* Intragenomic duplication, *GL* Gain or loss of mitochondrial sequences, *PH* Potential HDT, *NU* Nuclear genome derived, *ND* Not determined

Specifically, non-alignable sequences between *M. candidum* and *M. sanguineum* contain one region of 2.7 kb in *M. candidum* and two regions of totally 6.4 kb in *M. sanguineum*. Most of non-alignable sequences between the two species (95.39% in *M. candidum* and 84.14% in *M. sanguineum*) result from gain of short regions in one species or loss in the other. But loss of these regions in *M. candidum* or *M. sanguineum* is more phylogenetically parsimonious. Non-alignable sequences between *M. candidum* and *M. dodecandrum* contain 7.6 kb (seven regions) in *M. candidum* and 27.6 kb (16 regions) in *M. dodecandrum*. Most of the 7.6 kb in *M. candidum* (92.32%) are shared with *M. sanguineum*, suggesting gain of these short regions in the common ancestor of *M. candidum* and *M. sanguineum* or loss of them in *M. dodecandrum*. Most of the 27.6 kb in *M. dodecandrum* are chloroplast derived (31.62%) and potential horizontal DNA transfer (36.06%). Non-alignable sequences between *M. sanguineum* and *M. dodecandrum* contain 7.6 kb (seven regions) in *M. sanguineum* and 24.1 kb (16 regions) in *M. dodecandrum*. The origins of the non-alignable sequences between the two species are highly similar to those between *M. candidum* and *M. dodecandrum*.

Blastn searches of other non-alignable sequences against the nr database of GenBank are shown in Fig. 5. Some sequences got higher hit scores with mitochondrial sequences of non-Myrtales species, including some parasitic plants (*Aeginetia indica* and *Orobanche austrohispanica* from Orobanchaceae), rather than closely related species (*Medinilla magnifica* from Melastomataceae and *Eucalyptus grandis* from Myrtlaceae). This suggests that these sequences are putatively horizontally transferred from other distantly related plants after the divergence of *Melastoma* species. But the accurate donors of these sequences are unclear as the taxa matched in the database are insufficient, and/or the hits are too short, for phylogenetic analysis. The proportion of potential HDT sequences cover roughly one third of the non-alignable regions in *M. dodecandrum*, suggesting that HDT also plays an important role in mitogenome size changes between *M. dodecandrum* and two other species. The occurrence of HDT in *Melastoma* may be facilitated by physical contact with parasitic plants, as also observed in other plants [60–62].

There is a very small fraction of the non-alignable sequences in all three species with hits to their nuclear genomes. With no genes on these sequences, it is hard to define whether they are IDTs from mitogenome to nuclear genome or vice versa. Further Blast search of these sequences against the nr database of GenBank revealed that most of these sequences also got hits to mitogenomic sequences of other plants, suggesting that these sequences are probably IDTs from mitogenomes to nuclear genomes.

Mitogenome sequence alignment between *M. candidum* and *M. sanguineum* shows an inversion of about 150 kb between them (Fig. 4). This inversion contains 22 protein coding genes, 10 tRNAs, one rRNA and an IDT containing four transferred chloroplast gene copies (ψ*ndhB* (intact in *M. candidum*), ψ*ycf2*, ψ*rpoB* and *rpl16*). The IDT event should occur prior to the inversion event because both species have the same transferred chloroplast gene copies. There are 62 indels and 121 nucleotide substitutions between the mitogenomes of *M. candidum* and *M. sanguineum*. The sizes of indels range from 1 to 8 bp and all the indels are located either in the intergenic spacers or in the introns. The 121 nucleotide substitutions contain 80 transversions and 41 transitions. Most of them exist in the intergenic spacers, and only two are in the coding regions of mitochondrial genes (one in *rps13* and the other in *matR*; Table S5). The two nucleotide substitutions are both nonsynonymous. We then used the early diverged species of this genus, *M. dodecandrum*, to infer the ancestral status at these substitution positions. Among the 105 substitutions in the aligned regions of the three species, 52 substitutions occurred in *M. candidum* and 53 in *M. sanguineum*. The only two substitutions in the coding regions contain one substitution in *matR* occurring in *M. candidum* and the other in *rps13* occurring in *M. sanguineum*.

Because the only two substitutions in mitochondrial coding regions between *M. candidum* and *M. sanguineum* are both nonsynonymous, we calculated the synonymous nucleotide substitution rate only between *M. candidum* and *M. dodecandrum*. More than 380 kb sequences could be aligned between the two species and totally 1751 nucleotide substitutions were found, among which only 27 were located in protein-coding regions (Table S5), and the synonymous substitution rate was 1.33 × 10^–3^ per site. The synonymous nucleotide substitution rate between *M. candidum* and *Eucalyptus* is 6.28 × 10^–2^ per site. Using a divergence time of 88 Ma for *Melastoma* and *Eucalyptus* (http://timetree.org/), we can estimate the divergence time between the two *Melastoma* species is 1.86 Ma, which is similar to a previous study based on chloroplast *ndhF* sequences [35].

There are 1624 nucleotide substitutions and 275 indels in non-coding regions between *M. candidum* and *M. sanguineum*, leading to a substitution rate of 4.45 × 10^–3^ per site. Based on the divergence time of 1.86 Ma, the synonymous substitution rate in coding regions is 7.15 × 10^–10^ per site per year, and the substitution rate in mitochondrial noncoding regions is 2.39 × 10^–9^ per site per year. The substitution rate in protein-coding genes is three-fold lower than that in non-coding sequences.

### A recurrent IDT event identified in a species of *Melastoma*

**Fig. 6:**
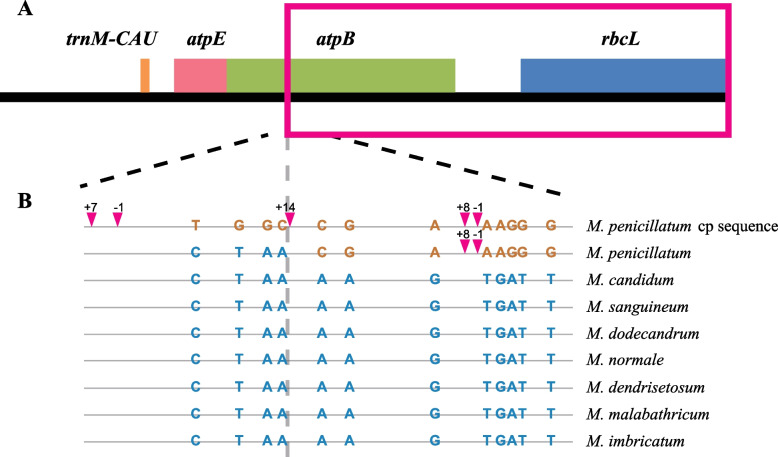
Schematic diagram of the recurrent IDT region in the mitogenomes of *Melastoma*. **A** Graphic display of the transferred *rbcL*-*atpB-atpE*-*trnM-CAU* region. The red box shows the recurrent IDT region. **B** Variable sites in the amplified ~ 600 bp fragments spanning the boundary of the recurrent IDT region. The corresponding chloroplast (cp) sequence of *M. penicillatum* was used for comparison. The inverted triangles and colored bases stand for indels (“ + ”: insertion; “-”: deletion) and nucleotide substitutions between the IDT region of *Melastoma* species and the corresponding region in the chloroplast genome. The numbers above the inverted triangles mean the lengths (bp) of indels. The grey vertical dashed line is the boundary between the original and recurrent IDTs

We amplified and sequenced a shared 5.1 kb IDT region in the three mitogenomes, which contained four chloroplast-derived pseudogenes, ψ*rbcL*, ψ*atpB*, ψ*atpE* and ψ*trnM-CAU*, from five other species of *Melastoma* in China (Fig. 6). PCR amplification showed that all eight species investigated in this study have this IDT event, suggesting that this event occurred in the common ancestor of these species. In *M. candidum*, there are 123 nucleotide substitutions and 44 indels between this IDT region in the mitogenome and the counterpart in its chloroplast genome. As annotated in the mitogenomes of the three species, the four genes are all pseudogenized in all but one species. The only one exception is *M. penicillatum* sampled from Jianfengling, in which most of this IDT region (3.3 kb) was identical in sequence to the corresponding region in the *M. candidum* chloroplast genome rather than being highly similar to this IDT region of other *Melastoma* species (Fig. 6). On the contrary, 1.8 kb at the other end of this IDT region in *M. penicillatum* matched well with those of other *Melastoma* species in sequence rather than its counterpart in the *M. candidum* chloroplast genome. Specifically speaking, the individual of *M. penicillatum* has nearly complete *rbcL* gene and most part of *atpB* gene, which indicated that a recurrent IDT from chloroplast to mitochondria has happened in this region. In this recurrent IDT region, there are 66 nucleotide substitutions and 16 indels in seven other species, compared with the sequence of *M. penicillatum* and the corresponding chloroplast sequence of *M. candidum*. This suggests that a recurrent IDT event occurred very recently within the initial IDT region in *M. penicillatum* so that no mutation has been accumulated in the recurrent IDT region.

**Fig. 7:**
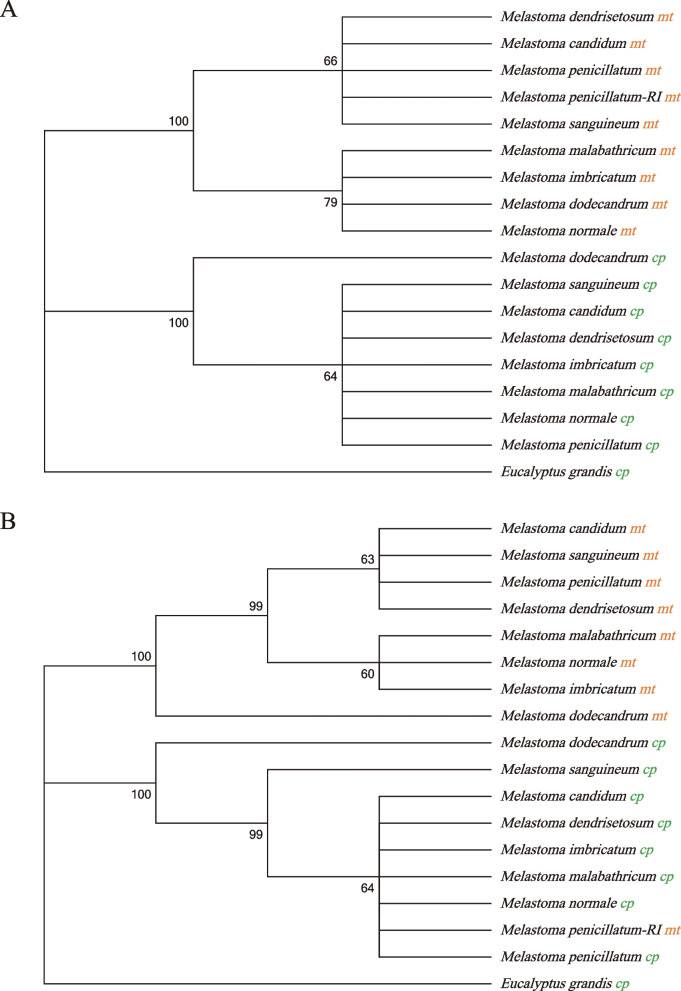
Maximum likelihood tree based on the transferred IDT sequences and the corresponding chloroplast sequences of multiple species in *Melastoma*. The letters *mt* indicates mitochondrial sequences, *cp* stands for chloroplast sequences, and RI stands for the individuals from Jianfengling with the recurrent IDT. Shown at the nodes are bootstrap support values. **A** ML tree based on the whole IDT region but excluding the recurrent IDT; **B** ML tree based on the recurrent IDT region

We further verified this recurrent IDT event by phylogenetic analysis based on sequences of this IDT region and the corresponding region in the chloroplast genome from multiple species of *Melastoma* (Fig. 7)*.* To avoid the influence of different origins, we divided the whole IDT region into two parts: the recurrent IDT region and the rest, and then constructed the maximum likelihood tree for them separately. It showed that the recurrent IDT region of *M. penicillatum* clustered with its chloroplast counterpart rather than with the IDT regions in other species of *Melastoma*, while for the rest *M. penicillatum* clustered with other species of *Melastoma*. Our study demonstrates that IDT can occur on the same genomic location repeatedly.

It is very common that chloroplast DNA fragments are transferred into mitochondrial genomes in plants, but reports on recurrent IDT replacing the initial IDT are very rare. A previous study inferred at least five independent intracellular transfers of chloroplast *rbcL* gene into the mitogenomes during angiosperm evolution, and suggested that the *rbcL* transfer to mitogenomes might have occurred hundreds of times [63]. Our findings, on the other hand, indicate that the transfer of chloroplast DNA to mitogenome is repeatable, even in a relatively short time. The occurrence of recurrent IDT or even multiple replacement of the original IDT should not be very rare in that recombination is easier for homologous regions [64].

Because this recurrent IDT event occurred very recently, we wonder whether it has been fixed in this species or not by characterizing it in five individuals from three populations, Diaoluoshan, Wuzhishan and Jianfengling. Our results showed that the recurrent IDT event was present in all five individuals from Jianfengling, but absent in all the samples from Diaoluoshan and Wuzhishan, indicating that this event has not been fixed in *M. penicillatum*.

As revealed in this study, the same mitochondrial region in different species of the same genus can have different origins. It is obvious that this kind of regions in the mitogenome is not suitable for reconstructing phylogenetic relationships among species, genera, and even families. Even for other mitochondrial regions that are not chloroplast derived, it should also be cautious because mitochondrial genome can incorporate foreign DNA from other plants [65, 66] and even fungi [67] by mechanisms such as illegitimate pollination, cell–cell contact and external vectors [17, 68]. In addition, chloroplast *rbcL* is one of the most widely used DNA barcodes in plants [69–71], and the transfer of chloroplast *rbcL* to mitogenomes in some plants can result in problems when amplifying this gene [72]. It is expected that both the chloroplast and mitochondrial copies of *rbcL* may be amplified or sometimes only the mitochondrial copy was amplified during PCR amplification for plants with transferred *rbcL* gene. Previous studies on the suspicious pseudogenization of *rbcL* gene in photosynthetic plants are likely the consequence of *rbcL* gene transfer, such as observed in *Beaumontia* [73], *Canella* [74], *Humbertia* [75], *Ipomoea* [76] and *Galphimia* [77]. Thus, there should be a caveat for species identification or phylogenetic analysis when using *rbcL* or other chloroplast region as a DNA barcode in plants because of potential chloroplast gene transfer.

## Conclusion

In this study, we assembled and annotated the mitogenomes of three *Melastoma* species and found that mitogenome size variations in *Melastoma* mainly result from mitochondrial sequence gain/loss, IDT and potential HDT. In addition, a recurrent IDT from chloroplast genome has occurred in a population of *M. penicillatum* but not fixed in this species, suggesting that the process of recurrent IDT is still ongoing. By characterizing the mitochondrial genome sequences of *Melastoma*, our study not only helps understand mitogenome size evolution in closely related species, but also cautions different evolutionary histories of mitochondrial regions due to potential recurrent IDT events in some populations or species.

## Supplementary Information

**Additional file 1: Table S1. **Sample information of *Melastoma *species used in this study.

**Additional file 2:** **Table S2. **Primers used for PCR amplification and sequencing.

**Additional file 3:** **Table S3. **Intron contents in the mitogenomes of three *Melastoma *species.

**Additional file 4: Table S4. **Frequencies of recombination mediated by repeats larger than 100 bp in the mitogenomes of three *Melastoma *species.

**Additional file 5:** **Table S5. **Synonymous (Syn) and Non-Synonymous (Non) nucleotide substitutions in the mitochondrial genes between *Melastoma candidum *and *M. sanguineum*, and between *M. candidum *and *M. dodecandrum*.

**Additional file 6:** **Fig. S1. **Morphological illustrations for *Melastoma candidum *(A), *M. sanguineum *(B), and *M. dodecandrum *(C).

**Additional file 7:** **Fig. S2. **Procedure of inferring the origins of non-alignable regions in the mitogenomes of *Melastoma.*

**Additional file 8:** **Fig. S3. **Gene map of the *Melastoma sanguineum *mitogenome. Chloroplast genome derived genes were not shown in this figure. Pseudogenes are marked with “Ψ”.

**Additional file 9:** **Fig. S4. **Gene map of the *Melastoma dodecandrum *mitogenome. Chloroplast genome derived genes were not shown in this figure. Pseudogenes are marked with “Ψ”.

**Additional file 10:** **Fig. S5. **Intracellular transferred DNA from chloroplast genome to the mitochondrial genome of *Melastoma sanguineum*. Chloroplast genome (one of the inverted repeats (IR) excluded here) is colored in green and mitochondrial genome is in orange with annotation information. Chloroplast-derived genes are marked in green and pseudogenes are marked with “Ψ”. Pale blue lines within the circle represent transferred regions between the two genomes.

**Additional file 11:** **Fig. S6. **Intracellular transferred DNA from chloroplast genome to the mitochondrial genome of *Melastoma dodecandrum*. Chloroplast genome (one of the IRs excluded here) is colored in green and mitochondrial genome is in orange with annotation information. Chloroplast-derived genes are marked in green and pseudogenes are marked with “Ψ”. Pale blue lines within the circle represent transferred regions from the chloroplast genome. “*” stand for transferred chloroplast genes that only appear in *M. dodecandrum*.

## Data Availability

Illumina and PacBio raw sequence reads of *M. candidum*, *M. sanguineum* and *M. dodecandrum* have been deposit in NCBI SRA database (https://www.ncbi.nlm.nih.gov/sra, Illumina reads accession numbers: SRR22574046, SRR23109727 and SRR19183021; PacBio reads accession numbers: SRR22574047, SRR23109726 and SRR23112531). Mitogenome sequences and annotations of *M. candidum*, *M. sanguineum* and *M. dodecandrum* are available in NCBI GenBank (https://www.ncbi.nlm.nih.gov/genbank, Accession numbers: MZ490595, MZ490596 and MZ490597). The nuclear genomes of the three species were available in the website http://evolution.sysu.edu.cn/Sequences.html.

## Abbreviations

*CMS*: Cytoplasmic male sterility
*HDT*: Horizontal DNA transfer
*IDT*: Intracellular DNA transfer
*IR*: Inverted repeat
