# Supplementary material for: Factors contributing to mitogenome size variation and a recurrent intracellular DNA transfer in Melastoma
Source: BMC Genomics. 2023 Jul 1;24:370. doi: 10.1186/s12864-023-09488-x (PMC10315049; doi:10.1186/s12864-023-09488-x)
Supplement: Supplementary file 1 — Additional file 1: Table S1. Sample information of Melastoma species used in this study. [file 12864_2023_9488_MOESM1_ESM.pdf]

Table S1. Sample information of *Melastoma* species used in this study.

Species	Location	Sample size	Voucher
M. candidum	Bamen Bay, Wenchang, Hainan, China	1	Zhou2021001
M. sanguineum	Bajia, Yangchun, Guangdong, China	1	Zhou2021002
M. dodecandrum	Tonghe, Guangzhou, Guangdong, China	1	Zhou2021003
M. imbricatum	Nanxi, Hekou, Yunnan, China	1	Zhou2021004
M. dendrisetosum	Diaoluoshan, Lingshui, Hainan, China	1	Zhou2021005
M. normale	Tangjiawan, Zhuhai, Guangdong, China	1	Zhou2021006
M. malabathricum	Zhenlong, Guangzhou, Guangdong, China	1	Zhou2021007
	Diaoluoshan, Lingshui, Hainan, China	5	Zhou2021008-Zhou2021012
M. penicillatum	Wuzhishan, Qiongzhong, Hainan, China	5	Zhou2021013-Zhou2021017
	Jianfengling, Lechang, Hainan, China	5	Zhou2021018-Zhou2021022
