# Supplementary material for: Factors contributing to mitogenome size variation and a recurrent intracellular DNA transfer in Melastoma
Source: BMC Genomics. 2023 Jul 1;24:370. doi: 10.1186/s12864-023-09488-x (PMC10315049; doi:10.1186/s12864-023-09488-x)
Supplement: Supplementary file 2 — Additional file 2: Table S2. Primers used for PCR amplification and sequencing. [file 12864_2023_9488_MOESM2_ESM.pdf]

Table S2. Primers used for PCR amplification and sequencing.

Primer	Sequence (5'-3')	Used for
mt-F	TTTCGGTTTTCGCACTCTGT	PCR amplification
mt-R	GGGATTTTAGCACGGATATAAG	PCR amplification
cp-F	TTTTTGCATTGGGCTCTTTC	PCR amplification
cp-R	AAGTATTCGGCTCAATCCTTTTT	PCR amplification
Internal01-R	ACTCCCAATTCTCTGGCAAA	Sequnecing
Internal02-R	CATCGGTCCACACAGTTGTC	Sequnecing
Internal03-R	CCCGGGAAGATGCCTAATA	Sequnecing
Internal04-R	CTTCCGTTTTGTCCAAGCAG	Sequnecing
Internal05-R	CGGCGAATTAGATGGTCTTC	Sequnecing
Internal06-F	CCCGTGTCAATCACTTCCAT	Sequnecing
