# Supplementary material for: Factors contributing to mitogenome size variation and a recurrent intracellular DNA transfer in Melastoma
Source: BMC Genomics. 2023 Jul 1;24:370. doi: 10.1186/s12864-023-09488-x (PMC10315049; doi:10.1186/s12864-023-09488-x)
Supplement: Supplementary file 3 — Additional file 3: Table S3. Intron contents in the mitogenomes of three Melastoma species. [file 12864_2023_9488_MOESM3_ESM.pdf]

Table S3. Intron contents in the mitogenomes of three *Melastoma* species.

Gene	Intron	Type	Length (bp)		
			M. candidum	***M. sanguineum***	***M. dodecandrum***
ccmFC	intron1	*cis*	962	961	957
cox2	intron1	*cis*	1,106	1,106	1,106
nad1	intron1	*trans*	-	-	-
	intron2	*cis*	1,335	1,335	1,335
	intron3	*trans*	-	-	
	intron4	*cis*	3,091	3091	3,091
nad2	intron1	*cis*	1,006	1,006	1,006
	intron2	*trans*	-	-	
	intron3	*cis*	2,235	2,235	2,236
	intron4	*cis*	1,499	1,499	1,499
nad4	intron1	*cis*	1,440	1,440	1,440
	intron2	*cis*	3,744	3,742	3,765
	intron3	*cis*	2,268	2,268	2,268
nad5	intron1	*cis*	853	853	853
	intron2	*trans*	-	-	-
	intron3	*trans*	-	-	-
	intron4	*cis*	1,099	1,099	1,099
nad7	intron1	*cis*	913	913	912
	intron2	*cis*	1,032	1,032	1,032
	intron3	*cis*	976	976	975
	intron4	*cis*	1,687	1,687	1,687
rpl2	intron1	*cis*	1,505	1,505	1,505
rps3	intron1	*cis*	1,214	1,214	1,215
rps10	intron1	*cis*	789	789	789
