# Supplementary material for: Factors contributing to mitogenome size variation and a recurrent intracellular DNA transfer in Melastoma
Source: BMC Genomics. 2023 Jul 1;24:370. doi: 10.1186/s12864-023-09488-x (PMC10315049; doi:10.1186/s12864-023-09488-x)
Supplement: Supplementary file 4 — Additional file 4: Table S4. Frequencies of recombination mediated by repeats larger than 100 bp in the mitogenomes of three Melastoma species. [file 12864_2023_9488_MOESM4_ESM.pdf]

Table S4. Frequencies of recombination mediated by repeats larger than 100 bp in the mitogenomes of three *Melastoma* species.

M. candidum			***M. sanguineum***			***M. dodecandrum***		
Repeat Unit	Length (bp)	Recombination rate	Repeat Unit	Length (bp)	Recombination rate	Repeat Unit	Length (bp)	Recombination rate
Repeat_1	712	2.86%	Repeat_1	592	0.00%	Repeat_1	878	1.91%
Repeat_2	359	1.89%	Repeat_2	416	2.00%	Repeat_2	758	3.40%
Repeat_3	356	0.86%	Repeat_3	369	0.00%	Repeat_3	369	0.00%
Repeat_4	307	1.74%	Repeat_4	359	0.00%	Repeat_4	259	1.08%
Repeat_5	259	0.73%	Repeat_5	307	0.00%	Repeat_5	200	0.00%
Repeat_6	192	0.33%	Repeat_6	259	0.86%	Repeat_6	193	0.00%
Repeat_7	189	0.00%	Repeat_7	192	0.67%	Repeat_7	192	0.85%
Repeat_8	189	1.07%	Repeat_8	189	0.00%	Repeat_8	188	0.75%
Repeat_9	185	1.16%	Repeat_9	185	0.00%	Repeat_9	177	0.00%
Repeat_10	181	0.00%	Repeat_10	181	0.00%	Repeat_10	150	0.36%
Repeat_11	143	0.00%	Repeat_11	173	0.00%	Repeat_11	143	0.00%
Repeat_12	139	0.00%	Repeat_12	145	0.00%	Repeat_12	134	0.00%
Repeat_13	137	0.00%	Repeat_13	143	0.00%	Repeat_13	131	0.33%
Repeat_14	131	0.00%	Repeat_14	137	0.00%	Repeat_14	113	0.00%
Repeat_15	113	0.32%	Repeat_15	131	0.00%	Repeat_15	110	0.33%
Repeat_16	110	0.37%	Repeat_16	119	0.79%	Repeat_16	110	0.37%
Repeat_17	107	0.00%	Repeat_17	113	0.00%	Repeat_17	104	0.36%
			Repeat_18	110	0.00%			
			Repeat_19	107	0.00%			
