# Supplementary material for: Factors contributing to mitogenome size variation and a recurrent intracellular DNA transfer in Melastoma
Source: BMC Genomics. 2023 Jul 1;24:370. doi: 10.1186/s12864-023-09488-x (PMC10315049; doi:10.1186/s12864-023-09488-x)
Supplement: Supplementary file 5 — Additional file 5: Table S5. Synonymous (Syn) and Non-Synonymous (Non) nucleotide substitutions in the mitochondrial genes between Melastoma candidum and M. sanguineum, and between M. candidum and M. dodecandrum. [file 12864_2023_9488_MOESM5_ESM.pdf]

Table S5. Synonymous (Syn) and Non-Synonymous (Non) nucleotide substitutions in the mitochondrial genes between *Melastoma candidum* and *M. sanguineum*, and between *M. candidum* and *M. dodecandrum*.

Gene	Nucleotide position in the gene	Nucleotide in *M. candidum*	Nucleotide in *M. sanguineum*	Substitution type
rps13	243	A	C	Non
matR	671	C	T	Non

Gene	Nucleotide position in the gene	Nucleotide in *M. candidum*	Nucleotide in *M. dodecandrum*	Substitution type
atp1	517	C	A	Syn
atp1	1219	G	T	Non
atp8	255	C	A	Syn
ccmFC	212	A	C	Non
ccmFC	830	G	A	Non
ccmFN	1008	A	T	Non
ccmFN	1022	C	A	Non
matR	625	C	A	Non
matR	671	C	T	Non
matR	1360	A	C	Non
nad3	13	T	G	Non
nad4	90	G	T	Syn
nad4	246	G	T	Non
nad5	1731	C	A	Non
nad6	45	G	A	Syn
nad6	471	C	A	Syn
nad9	35	G	A	Non
nad9	110	A	C	Non
rpl2	1448	A	T	Syn
rpl2	1679	C	G	Syn
rpl5	144	C	A	Syn
rpl10	150	T	G	Non
rps1	463	A	G	Non
rps10	187	G	A	Non
rps10	337	A	C	Non
sdh3	227	T	C	Non
sdh4	186	C	A	Syn
