# Supplementary figures and images for: Factors contributing to mitogenome size variation and a recurrent intracellular DNA transfer in Melastoma
Source: BMC Genomics. 2023 Jul 1;24:370. doi: 10.1186/s12864-023-09488-x (PMC10315049; doi:10.1186/s12864-023-09488-x)

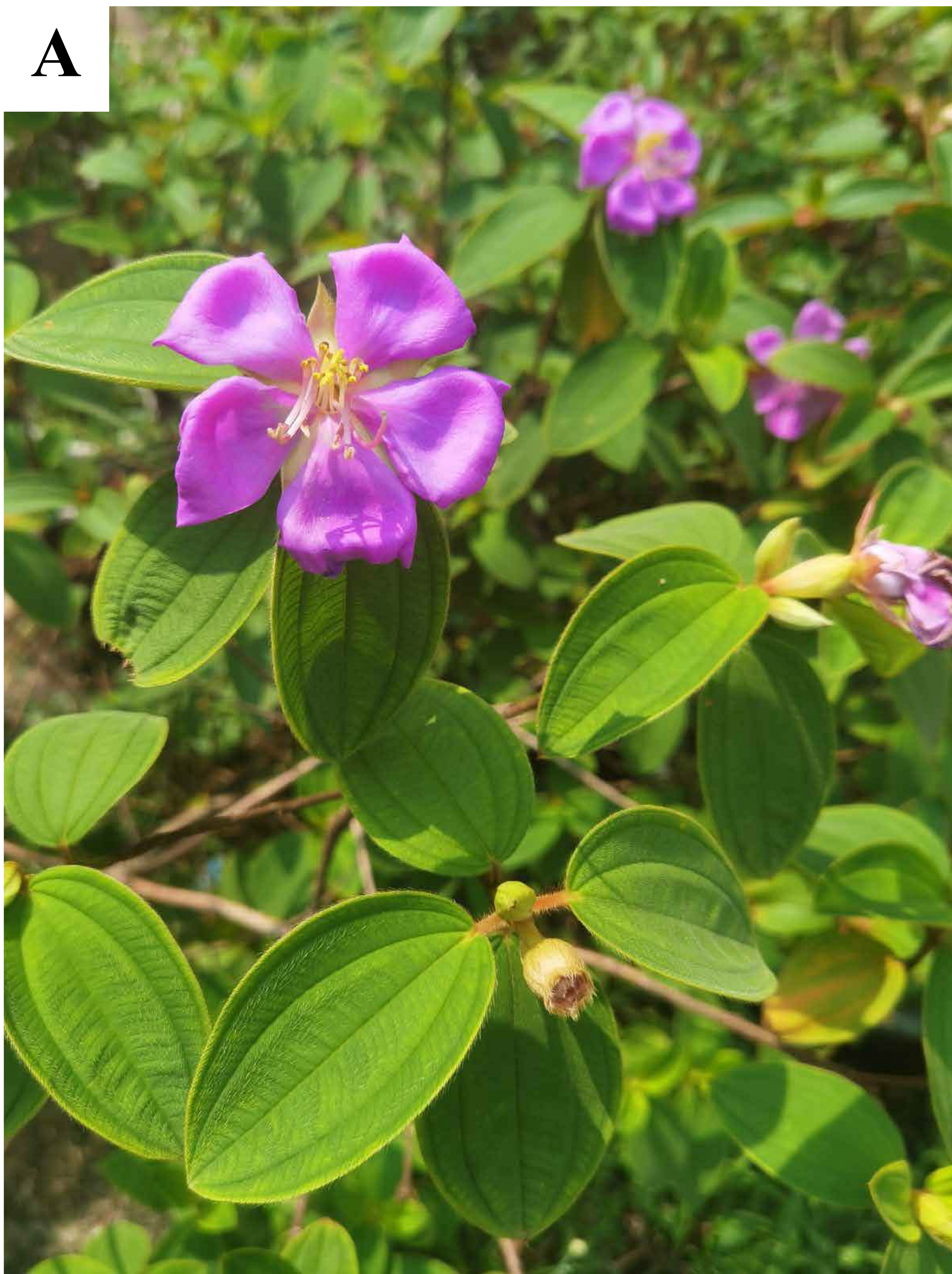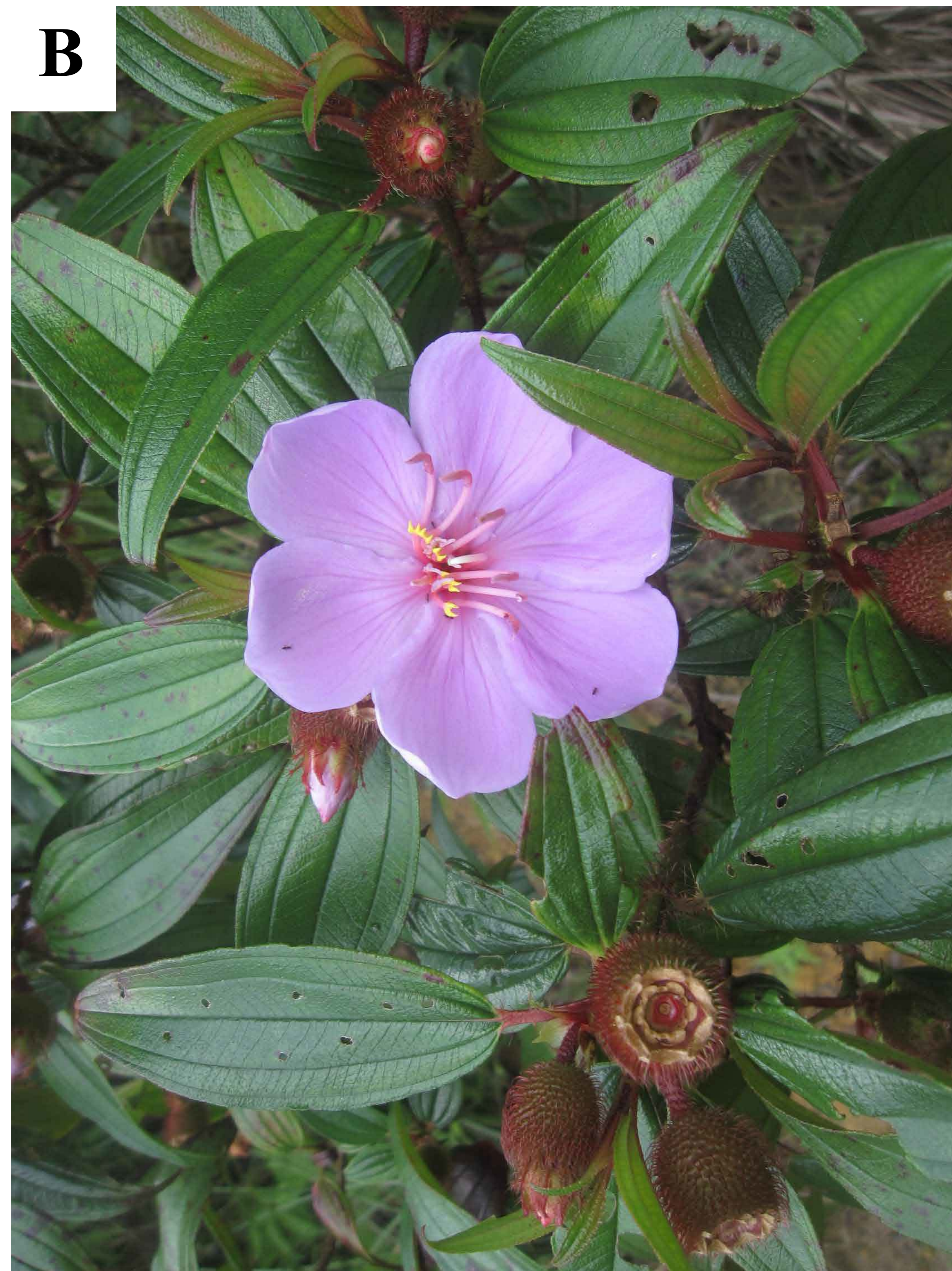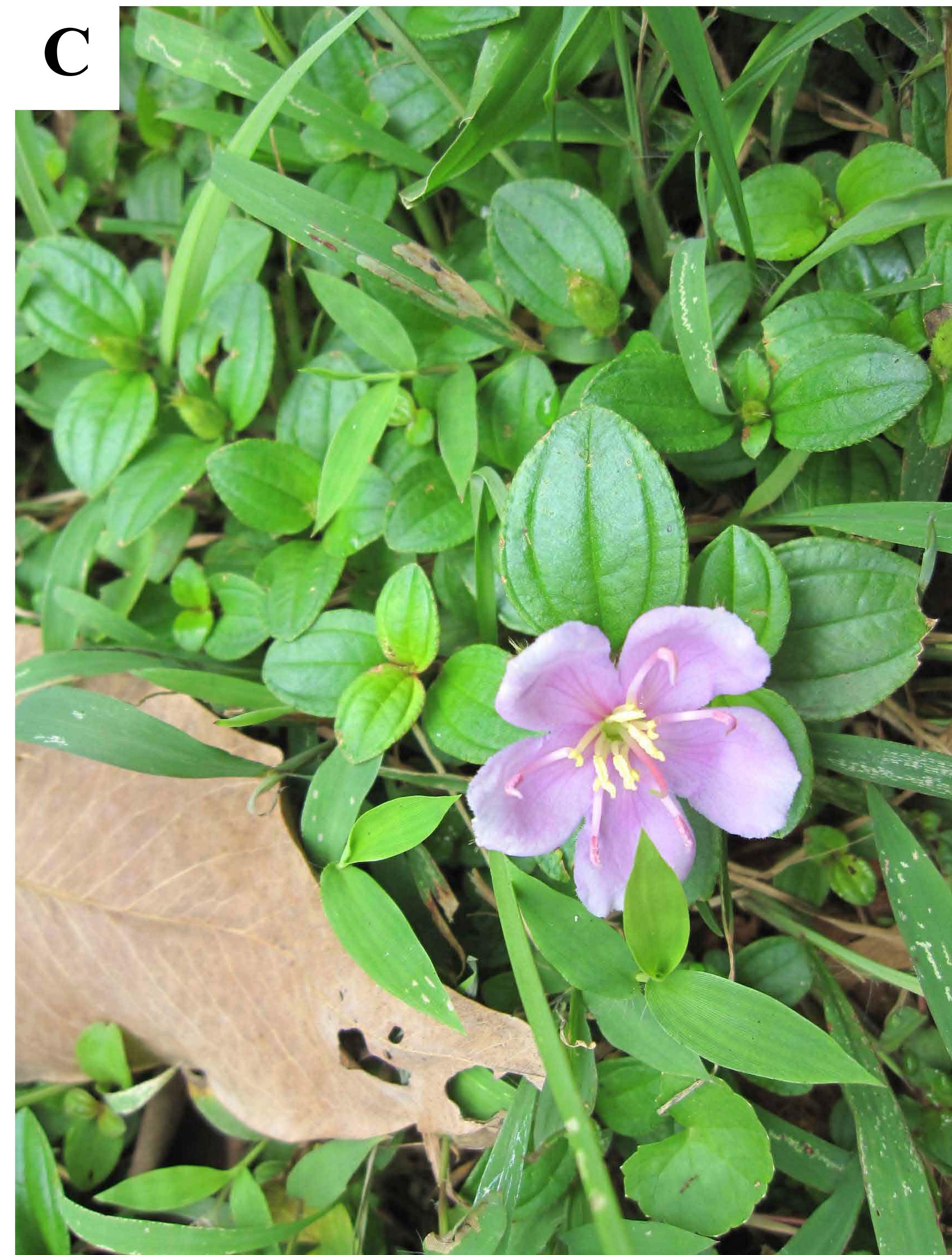

**Fig. S1.** Morphological illustrations for *Melastoma candidum* (A), *M. sanguineum* (B), and *M. dodecandrum* (C).

Supplement: Supplementary file 6 — Additional file 6: Fig. S1. Morphological illustrations for Melastoma candidum (A), M. sanguineum (B), and M. dodecandrum (C). [file 12864_2023_9488_MOESM6_ESM.pdf]

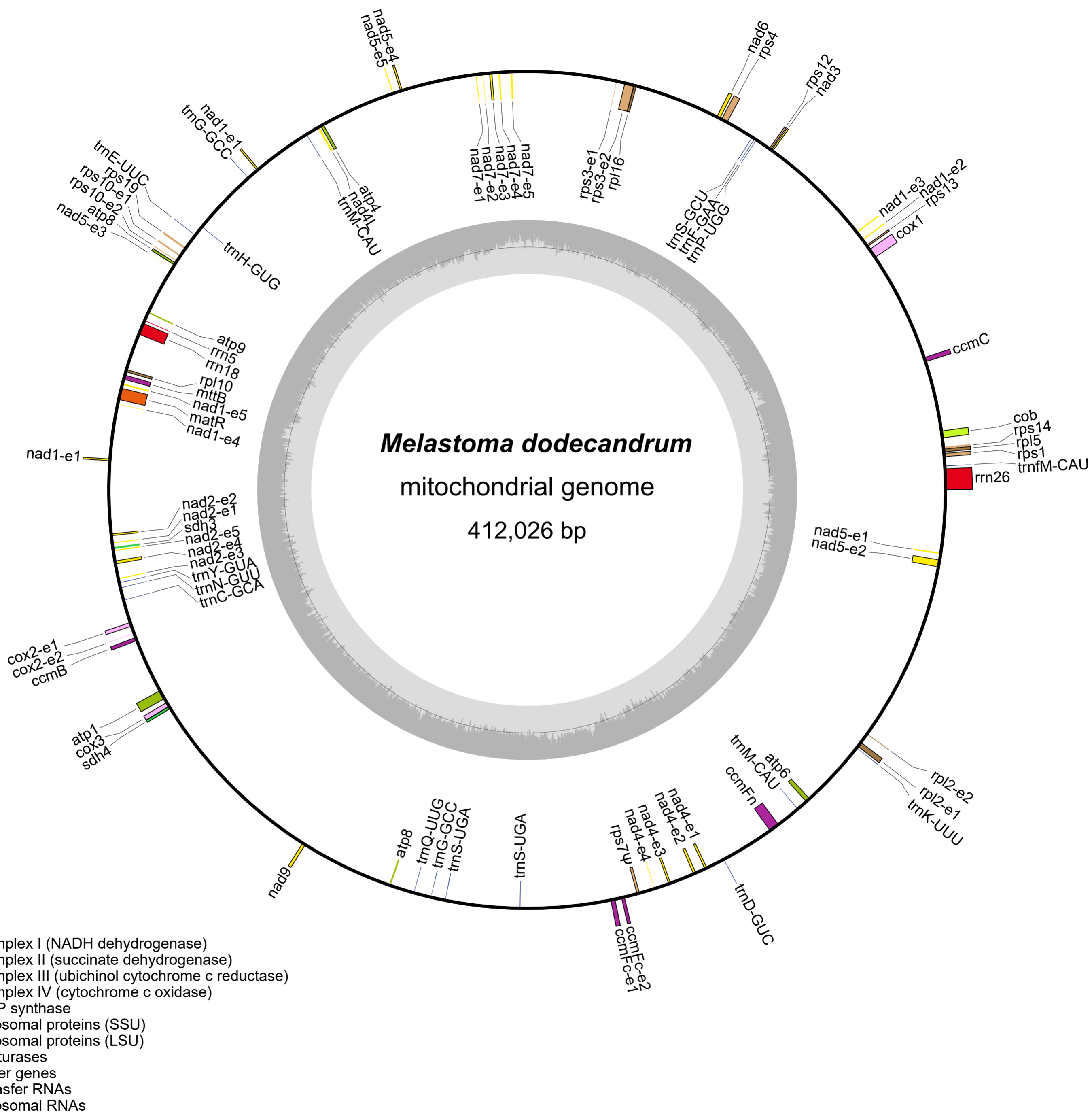

Supplement: Supplementary file 9 — Additional file 9: Fig. S4. Gene map of the Melastoma dodecandrum mitogenome. Chloroplast genome derived genes were not shown in this figure. Pseudogenes are marked with “Ψ”. [file 12864_2023_9488_MOESM9_ESM.pdf]
