# Supplementary material for: Factors contributing to mitogenome size variation and a recurrent intracellular DNA transfer in Melastoma
Source: BMC Genomics. 2023 Jul 1;24:370. doi: 10.1186/s12864-023-09488-x (PMC10315049; doi:10.1186/s12864-023-09488-x)
Supplement: Supplementary file 7 — Additional file 7: Fig. S2. Procedure of inferring the origins of non-alignable regions in the mitogenomes of Melastoma. [file 12864_2023_9488_MOESM7_ESM.pdf]

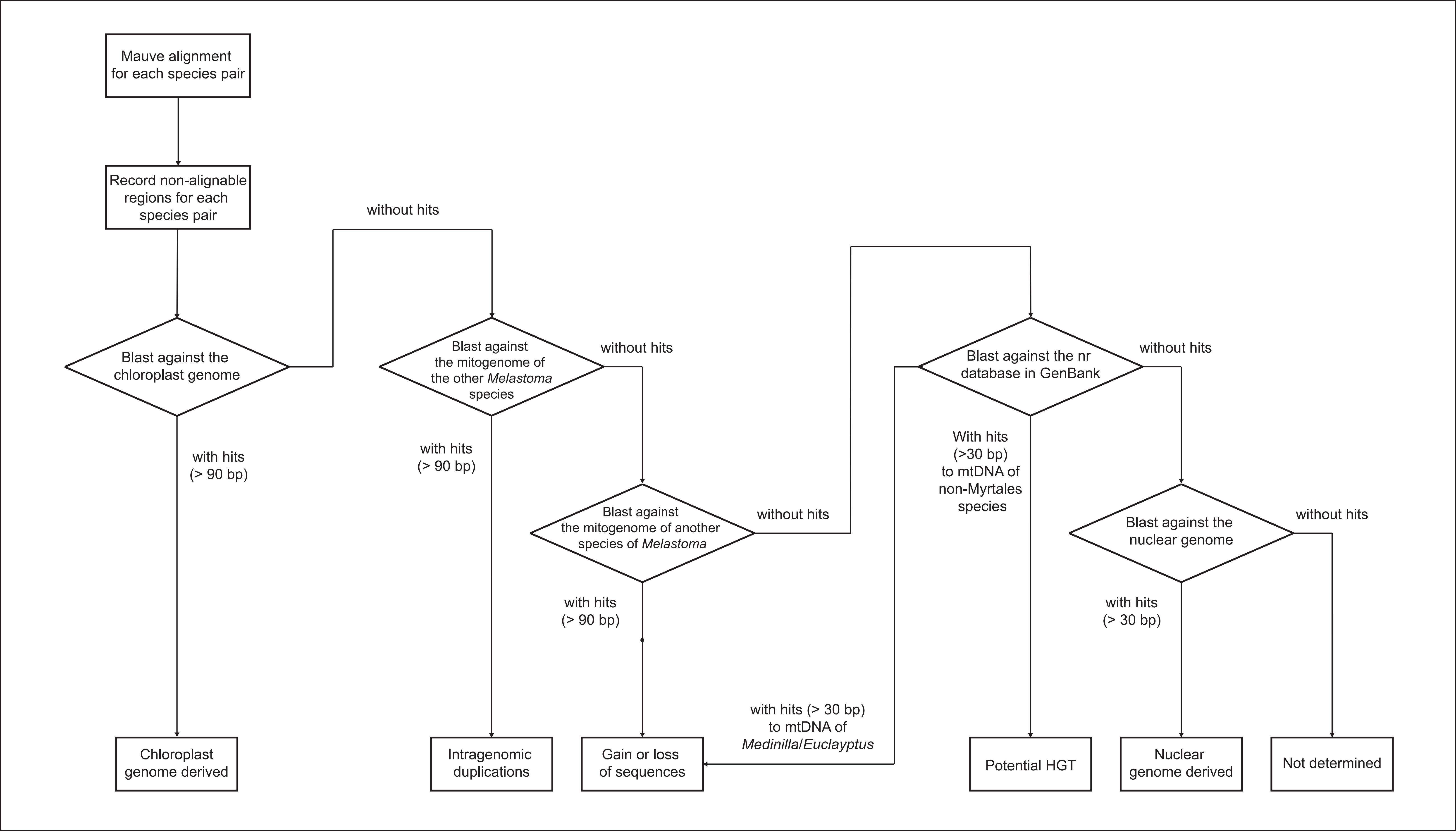

Fig. S2. Procedure of inferring the origins of non-alignable regions in the mitogenomes of *Melastoma.*
