# Supplementary material for: Factors contributing to mitogenome size variation and a recurrent intracellular DNA transfer in Melastoma
Source: BMC Genomics. 2023 Jul 1;24:370. doi: 10.1186/s12864-023-09488-x (PMC10315049; doi:10.1186/s12864-023-09488-x)
Supplement: Supplementary file 8 — Additional file 8: Fig. S3. Gene map of the Melastoma sanguineum mitogenome. Chloroplast genome derived genes were not shown in this figure. Pseudogenes are marked with “Ψ”. [file 12864_2023_9488_MOESM8_ESM.pdf]

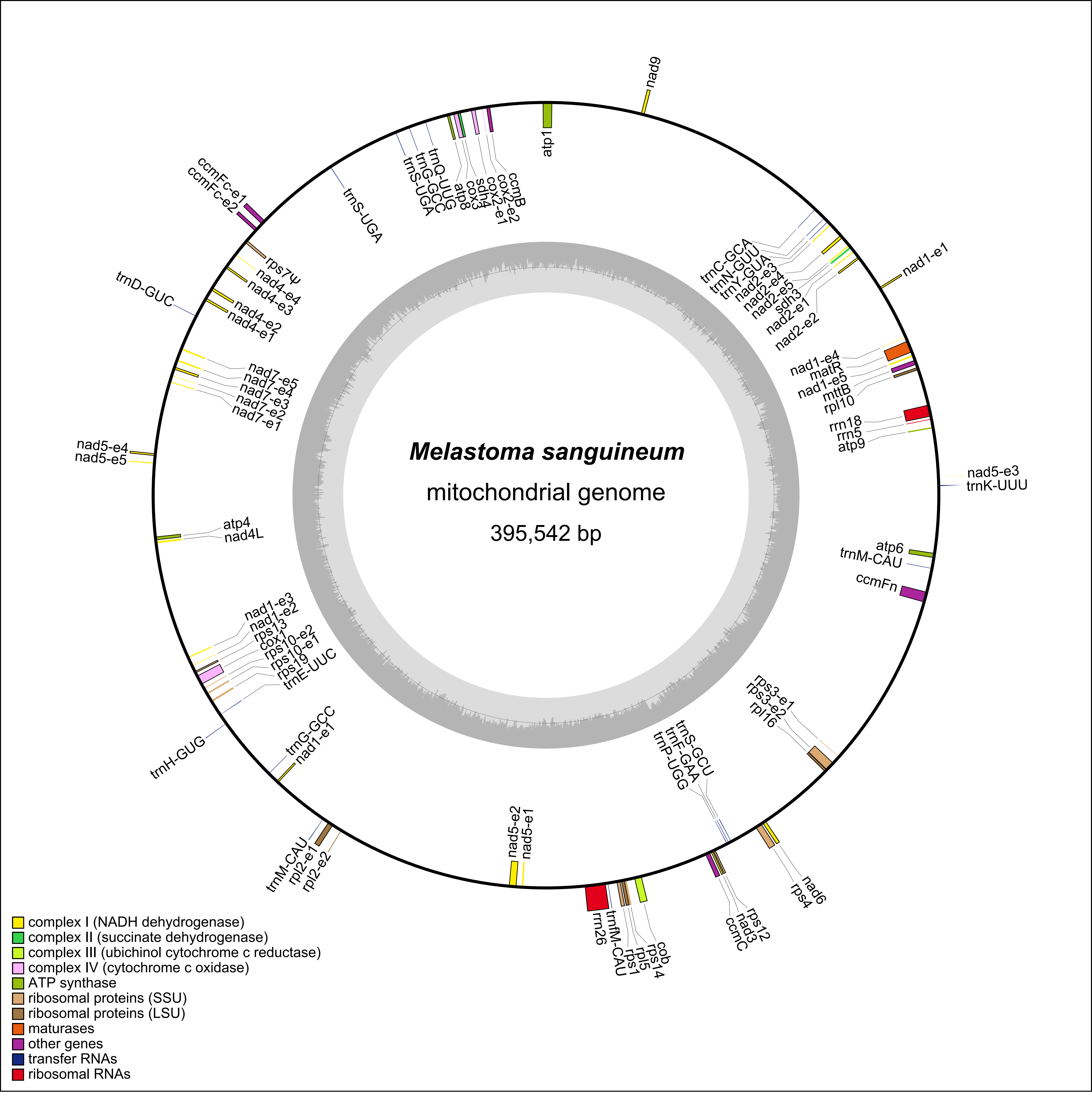

Fig. S3. Gene map of the *Melastoma sanguineum* mitogenome. Chloroplast genome derived genes were not shown in this figure. Pseudogenes are marked with “Ψ”.
