# Supplementary material for: Factors contributing to mitogenome size variation and a recurrent intracellular DNA transfer in Melastoma
Source: BMC Genomics. 2023 Jul 1;24:370. doi: 10.1186/s12864-023-09488-x (PMC10315049; doi:10.1186/s12864-023-09488-x)
Supplement: Supplementary file 10 — Additional file 10: Fig. S5. Intracellular transferred DNA from chloroplast genome to the mitochondrial genome of Melastoma sanguineum. Chloroplast genome (one of the inverted repeats (IR) excluded here) is colored in green and mitochondrial genome is in orange with annotation information. Chloroplast-derived genes are marked in green and pseudogenes are marked with “Ψ”. Pale blue lines within the circle represent transferred regions between the two genomes. [file 12864_2023_9488_MOESM10_ESM.pdf]

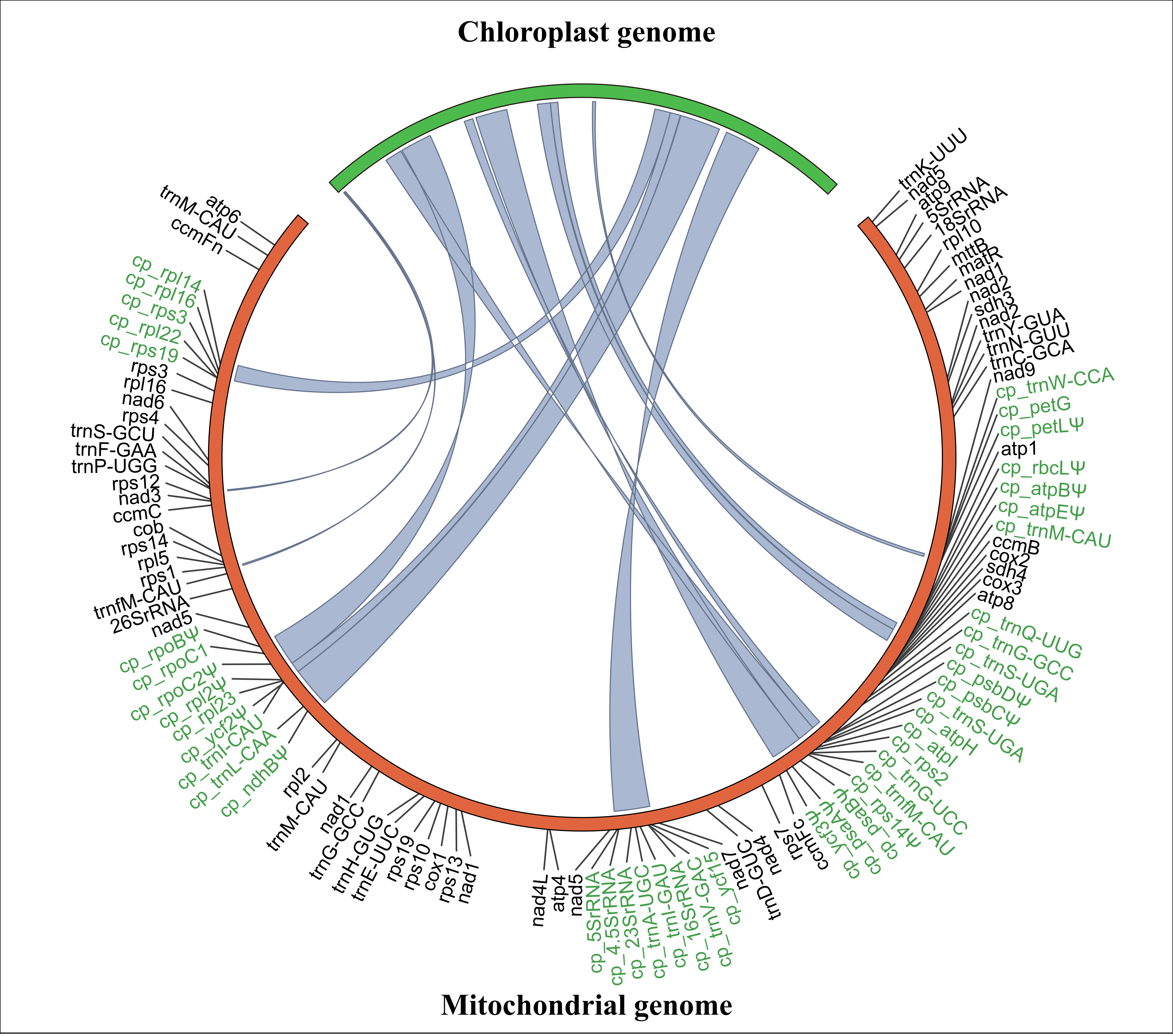

Fig. S5. Intracellular transferred DNA from chloroplast genome to the mitochondrial genome of *Melastoma sanguineum*. Chloroplast genome (one of the inverted repeats (IR) excluded here) is colored in green and mitochondrial genome is in orange with annotation information. Chloroplast-derived genes are marked in green and pseudogenes are marked with “Ψ”. Pale blue lines within the circle represent transferred regions between the two genomes.
