# Supplementary material for: Factors contributing to mitogenome size variation and a recurrent intracellular DNA transfer in Melastoma
Source: BMC Genomics. 2023 Jul 1;24:370. doi: 10.1186/s12864-023-09488-x (PMC10315049; doi:10.1186/s12864-023-09488-x)
Supplement: Supplementary file 11 — Additional file 11: Fig. S6. Intracellular transferred DNA from chloroplast genome to the mitochondrial genome of Melastoma dodecandrum. Chloroplast genome (one of the IRs excluded here) is colored in green and mitochondrial genome is in orange with annotation information. Chloroplast-derived genes are marked in green and pseudogenes are marked with “Ψ”. Pale blue lines within the circle represent transferred regions from the chloroplast genome. “*” stand for transferred chloroplast genes that only appear in M. dodecandrum. [file 12864_2023_9488_MOESM11_ESM.pdf]

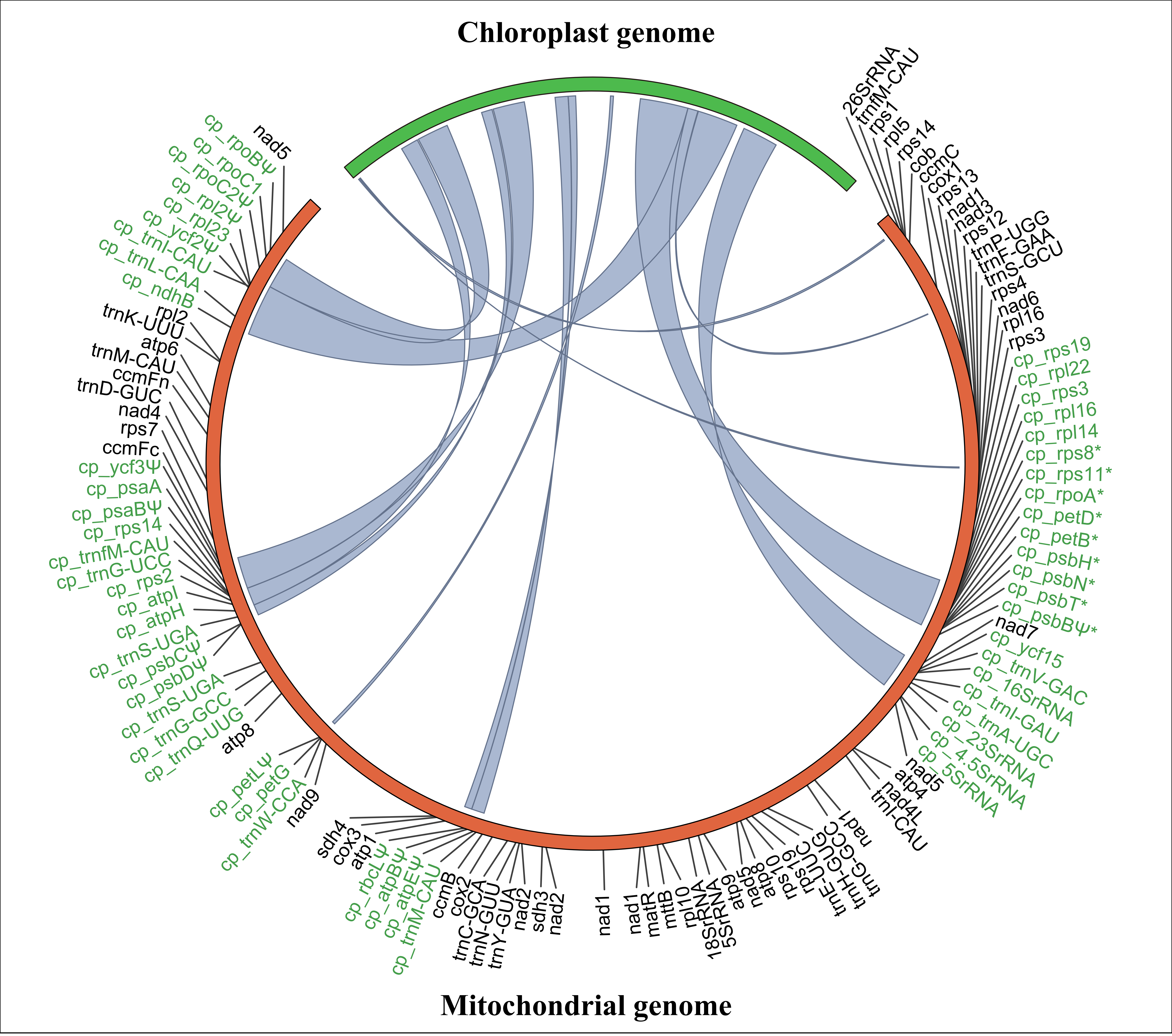

Fig. S6. Intracellular transferred DNA from chloroplast genome to the mitochondrial genome of *Melastoma dodecandrum*. Chloroplast genome (one of the IRs excluded here) is colored in green and mitochondrial genome is in orange with annotation information. Chloroplast-derived genes are marked in green and pseudogenes are marked with “Ψ”. Pale blue lines within the circle represent transferred regions from the chloroplast genome. “*” stand for transferred chloroplast genes that only appear in *M. dodecandrum*.
